# Partial Inhibition of Calcineurin Activity by Rcn2 as a Potential Remedy for Vps13 Deficiency

**DOI:** 10.3390/ijms22031193

**Published:** 2021-01-26

**Authors:** Patrycja Wardaszka, Piotr Soczewka, Marzena Sienko, Teresa Zoladek, Joanna Kaminska

**Affiliations:** Institute of Biochemistry and Biophysics Polish Academy of Science, 02-106 Warsaw, Poland; patrycja@ibb.waw.pl (P.W.); psoc@ibb.waw.pl (P.S.); marsi@ibb.waw.pl (M.S.); teresa@ibb.waw.pl (T.Z.)

**Keywords:** neurodegenerative diseases, calcium signaling, calcineurin, yeast model, Vps13, Rcn2

## Abstract

Regulation of calcineurin, a Ca^2+^/calmodulin-regulated phosphatase, is important for the nervous system, and its abnormal activity is associated with various pathologies, including neurodegenerative disorders. In yeast cells lacking the *VPS13* gene (*vps13*Δ), a model of *VPS13*-linked neurological diseases, we recently demonstrated that calcineurin is activated, and its downregulation reduces the negative effects associated with *vps13Δ* mutation. Here, we show that overexpression of the *RCN2* gene, which encodes a negative regulator of calcineurin, is beneficial for *vps13Δ* cells. We studied the molecular mechanism underlying this effect through site-directed mutagenesis of *RCN2*. The interaction of the resulting Rcn2 variants with a MAPK kinase, Slt2, and subunits of calcineurin was tested. We show that Rcn2 binds preferentially to Cmp2, one of two alternative catalytic subunits of calcineurin, and partially inhibits calcineurin. Rcn2 ability to bind to and reduce the activity of calcineurin was important for the suppression. The binding of Rcn2 to Cmp2 requires two motifs in Rcn2: the previously characterized C-terminal motif and a new N-terminal motif that was discovered in this study. Altogether, our findings can help to better understand calcineurin regulation and to develop new therapeutic strategies against neurodegenerative diseases based on modulation of the activity of selected calcineurin isoforms.

## 1. Introduction

Calcium ions (Ca^2+^) are important signaling molecules that regulate multiple processes. In neurons, calcium signaling is crucial for neurotransmitter release and for triggering a wide range of changes in neuronal function. Among the different proteins involved are Ca^2+^ sensors that transduce signals to specific effectors in a cell. One of the best-studied Ca^2+^-responsive signaling pathways involves a Ca^2+^/calmodulin-dependent protein phosphatase, calcineurin, which is also known to be important for the immune response [1]. Calcineurin is present in virtually all organisms. The architecture of this phosphatase, which is composed of catalytic and regulatory subunits, is conserved among species. In human cells, there are three different isoforms of the catalytic subunit (α, β, and γ, encoded by *PPP3CA*, *PPP3CB*, and *PPP3CC* genes, respectively) and two regulatory subunits (B1 and B2, encoded by *PPP3R1* and *PPP3R2* genes, respectively) [2,3]. The activity of calcineurin must be tightly regulated because its abnormal activity is associated with various pathologies in humans, including neurological and psychological disorders [4]. Therefore, calcineurin exists in an autoinhibited state and is activated when Ca^2+^ concentration is increased, and Ca^2+^/calmodulin is bound [5]. Thus, calcineurin inhibition may occur through Ca^2+^ or calmodulin sequestration or, alternatively, by blocking the binding of calmodulin to substrates or activators. The calcineurin recognizes two types of linear motifs in its substrates and regulators, PxIxIT and LxVP, by two binding pockets—one for each motif. PxIxIT-type motif recognition is independent of the presence of the regulatory subunit. The LxVP-type motif binds at the junction of catalytic and regulatory subunits [6]. Thus, blocking the pocket responsible for PxIxIT-type motif binding prevents the interaction of calcineurin with substrates that possess this type of motif without blocking calcineurin activity. By contrast, blocking the binding site for the LxVP-type motif not only interferes with substrate binding but also inhibits calcineurin activity. This site is also used by the immunosuppressants FK506 and cyclosporin A, which block substrate binding to the active p site of phosphatase [6]. They are common in medical use, although they have serious side effects [7,8]. There is a group of proteins, known as the regulators of calcineurin (RCANs) [9], that inhibit phosphatase activity by occupying substrate-binding motifs. In human cells, there are three RCAN-encoding genes, *RCAN1 (DSCR1/CALP1/MCIP1/ADAPT78*), *RCAN2* (*DSCR1L1/CALP2/ZAKI-4/MCIP2*), and *RCAN3* (*DSCR1L2⁄CALP3⁄MCIP3*) [10,11]. The most prominent feature identified in RCAN family proteins is a serine–proline motif (containing an ISPPXSPP box), which is conserved across species [9]. Moreover, at the N-terminus, weak homology with RNA-binding domains has been detected [10] and, recently, it was shown that this region of RCAN1 binds to RNA [12]. The human *RCAN1* gene has potential roles in Down syndrome, Alzheimer’s disease, in the regulation of the immune and inflammatory responses, and in different types of pathological angiogenesis [13]. All mammalian RCANs are expressed in the brain [10,13,14] and are important for brain physiology [15]. Furthermore, calcineurin catalytic subunits are specifically regulated by posttranslational modifications such as phosphorylation or ubiquitination [16,17].

In yeast cells, two alternative catalytic subunits of calcineurin are encoded by either *CNA1* or *CMP2/CNA2* genes [18], and only one regulatory subunit is encoded by *CNB1* [19]. There are also two RCANs: Rcn1 and Rcn2. The *RCN2* gene that encodes the Rcn2 protein was identified in a genetic screen for endogenous inhibitors of calcineurin [20]. Rcn2 shares structural and functional similarities to Rcn1 and RCAN proteins in other yeasts and is more distantly related to RCANs in mammals [9]. Active calcineurin dephosphorylates numerous factors that promote cell survival. In yeast, the best-investigated example is the transcription factor Crz1 [21], which is regulated similarly to the NFAT (nuclear factor of activated T cells) transcription factor in mammalian cells [6,22]. Furthermore, calcineurin inhibits a plasma membrane Ca^2+^ channel and a vacuolar H^+^/Ca^2+^ exchanger, both of which participate in the control cytosolic Ca^2+^ levels [23]. In yeast cells, proper regulation of calcineurin is important for the response to environmental stress, during which cytoplasmic Ca^2+^ levels are increased and for survival during long-term endoplasmic reticulum (ER) stress [24,25,26,27]. However, calcineurin is not important under standard growth conditions but becomes essential in cells lacking certain proteins involved in the maintenance of cell wall integrity, such as Slt2, Fks1, or Pkc1 [28]. Calcineurin activity is upregulated in yeast models of neurodegenerative diseases, including Parkinson’s disease [29]. Recently, we showed that calcineurin activity is increased in a yeast mutant lacking the Vps13 protein [30], indicating the link of Vps13 with calcium signaling.

The human genome contains four *VPS13* genes, *hVPS13A*-*D*, which encode proteins associated with the neurological disorders: chorea-acanthocytosis (ChAc) [31], Cohen syndrome [32], early-onset Parkinsonism [33], and spastic ataxia and paraplegia [34,35], respectively. *VPS13* genes are conserved among eukaryotes, and proteins of the Vps13 family show the highest homology in the N- and C-termini [36]. Vps13 proteins are proposed to be lipid transfer proteins based on in vitro studies showing that the N-terminus of yeast Vps13 is able to transfer lipids between liposomes [37]. This is also supported by a structural study of the N-terminal part of the Vps13 protein from *Chaetomium thermophilum* [38]. The high homology of the N-terminal part of all Vps13 proteins [36] suggests that they have a common function. However, the phenotypes caused by their dysfunction differ, which is indicative of differences in their regulation and/or localization. Indeed, differences in the localization of hVps13A and hVps13C proteins to membrane contact sites (MCS) have been described [37]. In yeast cells, the Vps13 protein localizes to various MCSs depending on the growth conditions [39,40,41]. Therefore, the localization of Vps13 proteins must be regulated by certain signals. Recently, it has been reported that the APT1 domains of hVps13A and yeast Vps13 bind signaling phosphoinositides and that this binding is differentially regulated by Ca^2+^ [42].

Noteworthy, a decrease in the level of Orai1 protein, the subunit of a channel responsible for Ca^2+^ entry into the cell, was found in fibroblasts of ChAc patients, resulting in a decrease in the influx of Ca^2+^ ions [43,44]. However, the synaptic hyperexcitability observed in ChAc neurons is not linked to changes in Ca^2+^ signaling but to changes in actin polymerization since actin influences the release of synaptic neurotransmitters [45]. More information about the function and regulation of Vps13 proteins, and the consequences of their defect, can be obtained using simple model organisms, such as yeast [46].

Since yeast cells have only one *VPS13* gene, which encodes a protein similar to all human Vps13 proteins, yeast can be used to study the effect of human *VPS13* gene mutations on cell physiology. This has already been successfully carried out to study the effect of mutations in *hVPS13A* and *hVPS13D* genes in terms of human diseases [30,40,47,48]. Recently, we demonstrated that in yeast cells lacking the *VPS13* gene (*vps13*Δ), the phosphorylation of MAPK kinase, the Slt2 protein, is increased and that calcium signaling is activated [30]. Moreover, we indicated that downregulation of this activity by Ca^2+^ chelation, administration of FK506, or calmodulin sequestration could mitigate the negative effects of *vps13*Δ mutation [30]. A more comprehensive understanding of the components, functions, and molecular mechanism of the Ca^2+^/calcineurin signaling cascade in yeast models of diseases will provide insights into pathological interactions and facilitate the development of novel therapeutic approaches.

In this work, we show that overexpression of the *RCN2* gene, which encodes a negative regulator of calcineurin, is beneficial for *vps13*Δ mutant cells, and we studied the molecular mechanisms underlying *RCN2* action. The results of site-directed mutagenesis, two-hybrid interaction studies, and phenotypic analysis show that suppression does not involve the interaction of Rcn2 protein with Slt2 kinase but involves binding with one of the calcineurin catalytic subunits, the Cmp2 protein. These results demonstrate that effective suppression of *vps13* mutant phenotypes can be obtained by inhibiting a subset of calcineurin complexes containing a particular isoform of a catalytic subunit, and this finding could be used in the development of new strategies to treat numerous neurodegenerative diseases.

## 2. Results

### 2.1. The RCN2 Gene is a Multicopy Suppressor of vps13Δ SDS Hypersensitivity

**Figure 1:**
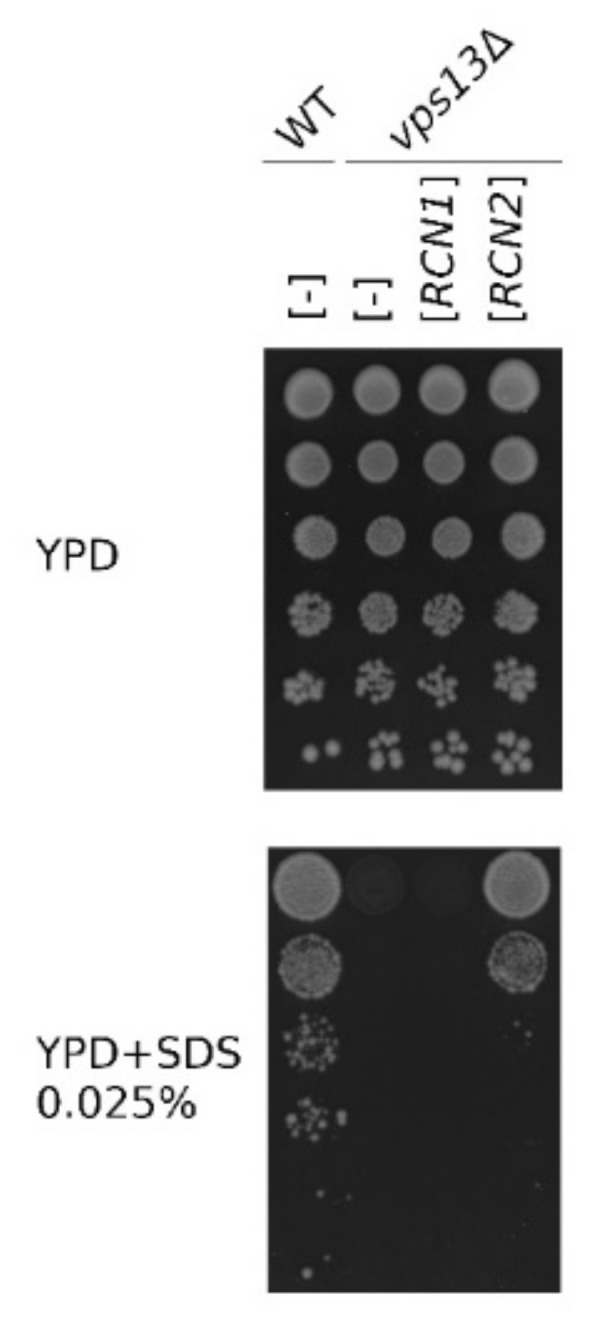
*RCN2*, but not *RCN1*, is a multicopy suppressor of the sodium dodecyl sulfate (SDS) hypersensitivity phenotype of the *vps13*Δ mutant. Serial dilutions of wild-type and *vps13*Δ strains transformed with empty vector [-] or vector bearing *RCN2* or *RCN1* genes were plated on YPD and YPD + SDS media. Plates were incubated for 3 days.

A *vps13* mutants exhibit several phenotypes: they show defects in protein transport [49,50,51], sporulation [52], and actin cytoskeleton organization [47]. Our studies indicate the alteration of calcium homeostasis in *vps13*Δ cells and a functional connection between Vps13 and calcium signaling [30]. To this list, we recently added a new phenotype: hypersensitivity to low concentrations of a commonly used amphiphilic detergent, sodium dodecyl sulfate (SDS), which we used in a genetic screen for multicopy suppressors of the *vps13-I2749R* mutation. This screen resulted in the identification of fragments of the *MYO3* gene (*MYO3-N*) and of the *FET4* gene [30,53] as suppressors, and both were also effective in *vps13*Δ cells. In this screen, we also found that a plasmid containing the *RCN2* gene, which encodes a negative regulator of calcineurin, restored the growth of the *vps13*Δ mutant in the presence of SDS (Figure 1). The paralogous *RCN1* gene, which encodes the other calcineurin regulator, has not been isolated; thus, we directly tested whether the overexpression of *RCN1* [54] could restore the growth of *vps13*Δ cells on SDS medium. In contrast to *RCN2,* overexpressed *RCN1* was not active as a suppressor (Figure 1). These results support the view that specific downregulation of calcium signaling, including that caused by Rcn2, is beneficial for *vps13*Δ mutant cells.

### 2.2. Binding of Rcn2 to Slt2 Kinase is Not Important for Suppression

**Figure 2:**
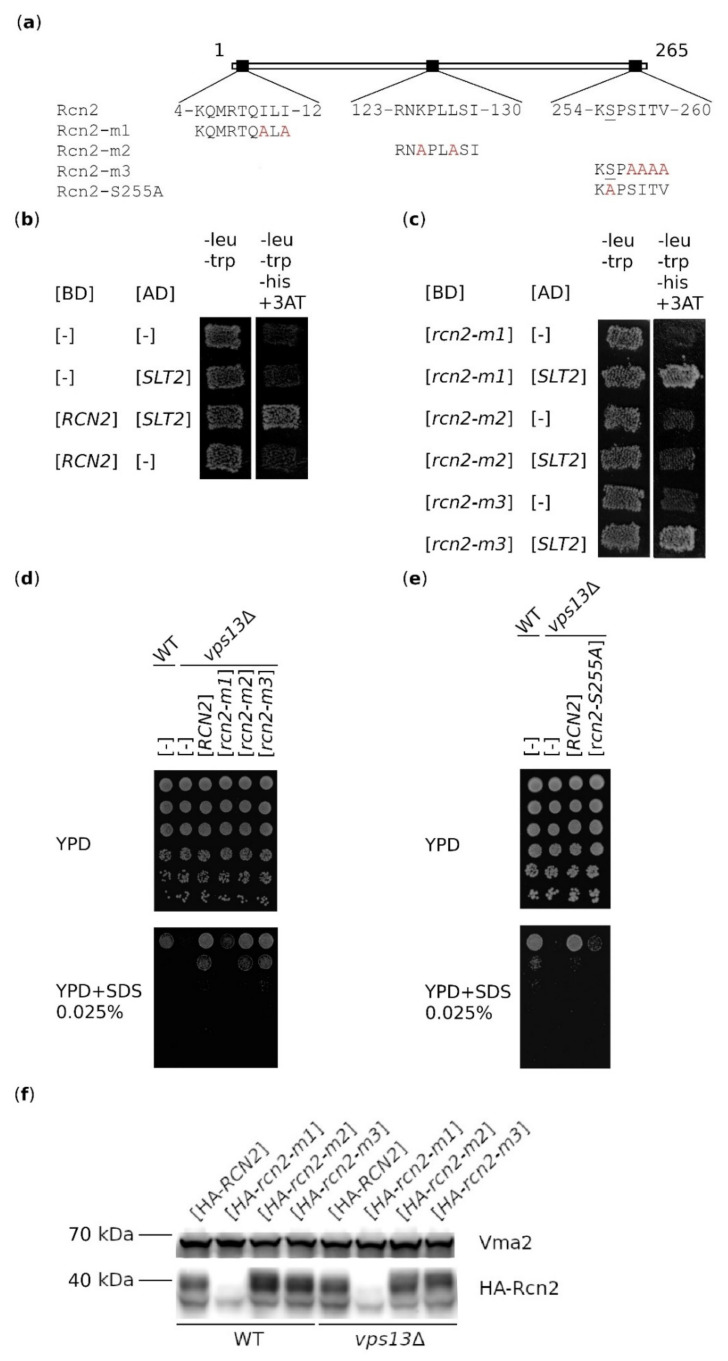
Rcn2-binding to Slt2 is not important for suppression of *vps13*Δ SDS hypersensitivity, in contrast to serine residue S255 and the motif at the N-terminus of Rcn2. (**a**) Schematic representation of Rcn2 indicating three potential Slt2-binding sites. Amino acid residues substituted in mutant proteins, Rcn2-m1, Rcn2-m2, Rcn2-m3, and Rcn2-S255A, are in red. S255 is underlined. (**b**) Rcn2 and Slt2 interact in the two-hybrid system. (**c**) Rcn2-m2 does not interact with Slt2. BD, DNA-binding domain; AD, activating domain; 3AT, 3-aminotriazole. *rcn2-m1* (**d**) and *rcn2-S255A* (**e**) are not able to suppress the hypersensitivity of *vps13*Δ to SDS. Serial dilution of wild-type and *vps13*Δ strains transformed with empty vector [-] or vector bearing the *RCN2* gene were plated on YPD and YPD + SDS. Plates were incubated for 3 days. (**f**) *rcn2-m1* is less efficiently expressed. Western blot analysis of HA-Rcn2 and HA-Rcn2-m1, -m2 and -m3 levels in wild-type and *vps13*Δ mutant cells. The tagged versions of Rcn2 were detected with the anti-HA antibody. The levels of vacuolar protein Vma2 were used as a loading control.

Due to the fact that Rcn2 is a substrate of Slt2 kinase [55] and that the *vps13*Δ mutant shows increased Slt2 activity upon SDS treatment [30], we speculated that the mechanism of suppression could rely on Rcn2–Slt2-binding and Rcn2 phosphorylation. To test this hypothesis, we evaluated the importance of Rcn2 interaction with Slt2 and specifically of serine amino acid residue (aa) S255, which is phosphorylated by Slt2 [55], in the *RCN2*-based suppression of *vps13*Δ. The consensus of the motif recognized by Slt2 kinase in its target proteins is (R/K)_1−2_ − (*X*)_2−6_ − Φ − *X* − Φ), where *X* denotes any aa and Φ denotes a hydrophobic aa [56]. There are three such motifs in Rcn2, which are potential Slt2-binding sites (Figure 2a). We analyzed the effects of *rcn2-m1, -m2* and *-m3* mutations, each of which causes 3–4 aa substitutions for alanine (Figure 2a). The interaction between Slt2 and wild-type or mutant Rcn2 proteins was tested using a two-hybrid system. As shown in Figure 2b, the co-expression of *RCN2* and *SLT2* resulted in restored growth on media without leucine, tryptophan and histidine, indicating the interaction of wild-type Rcn2 protein with Slt2, while the interaction was lost between Rcn2-m2 and Slt2. These substitutions did not result in the lack of expression of *BD-rcn2-m2* (Figure S1). This shows that aa substitutions in the Rcn2-m2 protein are in the region important for Slt2-binding.

The mutation causing substitution of the S255 residue, which is phosphorylated by Slt2, for alanine was previously shown to result in the greatest reduction in Rcn2 phosphorylation [55]. This mutation was, therefore, introduced into *RCN2* (Figure 2a). In the next step, we tested how all introduced mutations affect the *RCN2*-based suppression of *vps13*Δ SDS hypersensitivity. The *rcn2-m2* and *rcn2-m3* alleles were effective as suppressors (Figure 2d), although the protein encoded by *rcn2-m2* was not able to bind Slt2 (Figure 2c). By contrast, the *rcn2-m1* and *rcn2-S255A* alleles (Figure 2d,e) lost the ability to support the growth of the *vps13*Δ mutant when overexpressed. This result could be explained by the introduction of *rcn2-m* mutations causing instability of the mutant protein or abolishing Rcn2 function in a way that is important for its suppressor activity. To distinguish between these two possibilities, the cellular levels of hemagglutinin (HA)-tagged versions of wild-type and mutant Rcn2 proteins were compared. Previous studies of the *rcn2-S255A* mutation found that it did not affect the level of the Rcn2 protein, so this was not tested in our study [55]. As shown in Figure 2f, the HA-Rcn2 protein migrated as multiple bands, ranging from about 37 to 55 kDa, even though the estimated molecular mass of the tagged version is about 32.5 kDa. The tagged version HA-Rcn2 is probably not functional because its overproduction did not reverse the SDS sensitivity of the *vps13*Δ mutant (Figure S2). The observed slower migrating bands could include forms that are post-translationally modified (phosphorylated, ubiquitinated) to mark HA-Rcn2 for degradation. The Western blot analysis revealed that the HA-Rcn2-m1 protein is produced but at a low level. In the case of HA-Rcn2-m2, the pattern of bands was slightly different compared with that of HA-Rcn2 and HA-Rcn-m3. Thus, it is possible that a lack of Slt2-binding affects Rcn2 phosphorylation and/or other posttranslational modifications. The level and modification pattern of the HA-Rcn2-m3 protein were similar to those of HA-Rcn2. These results indicate that HA-Rcn2 is a highly modified protein and that the N-terminal part of Rcn2 is important for its stability and functionality. In summary, these results show that the lack of interaction between Slt2 and Rcn2 does not interfere with the suppression of *vps13*Δ mutant phenotypes, but the presence of the S255 residue and, therefore, phosphorylation of this residue is necessary. The inability of *rcn2-m1* to suppress *vps13*Δ could be due to a low level of Rcn2-m1, but it is also possible that the N-terminal motif of Rcn2 containing isoleucine residues I10 and I12 is specifically important.

Since the mechanism of suppression is not based on Slt2–Rcn2 interaction, we considered the second possibility that Rcn2 acts as a negative regulator of calcineurin and that downregulation of this phosphatase activity is the basis for suppression.

### 2.3. The Suppression of vps13Δ by RCN2 Requires Calcineurin

**Figure 3:**
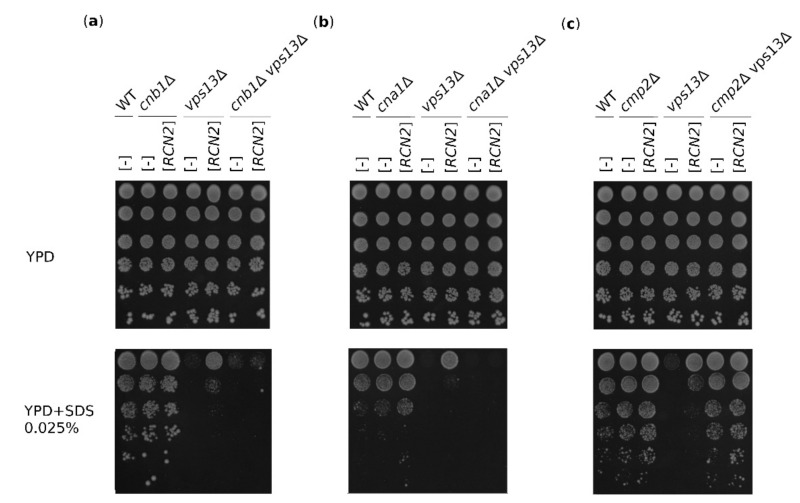
The suppression of *vps13*Δ mutant SDS hypersensitivity by *RCN2* requires active calcineurin. The growth of wild-type and *cnb1*Δ, *vps13*Δ, and *cnb1*Δ *vps13*Δ mutants (**a**), wild-type, *cna1*Δ, *vps13*Δ, and *cna1*Δ *vps13*Δ (**b**), and wild-type, *cmp2*Δ, *vps13*Δ, and *cmp21*Δ *vps13*Δ mutants (**c**) transformed with empty vector [-] or vector bearing the *RCN2* gene on YPD and YPD + SDS medium. Plates were incubated for 3 days.

RCANs are also involved in calcineurin-independent processes, such as binding mRNA and affecting its stability [12,13], so we tested whether Rcn2-based suppression requires calcineurin presence and/or activity. For this reason, the double mutants *cnb1*Δ *vps13*Δ, *cna1*Δ *vps13*Δ, and *cmp2*Δ *vps13*Δ were constructed that lacked either a regulatory (Cnb1) or catalytic (Cna1 or Cmp2) subunit. These mutants were transformed with an *RCN2*-bearing plasmid, and their growth on SDS-containing media was tested. The double *cnb1*Δ *vps13*Δ mutant was hypersensitive to SDS, and *RCN2* overexpression did not cause suppression (Figure 3a). Similarly, in the *cna1*Δ *vps13*Δ double mutant, there was no *RCN2*-based suppression (Figure 3b). Because the phosphatase is inactive in the *cnb1*Δ strain [19], the lack of suppression in the *cnb1*Δ *vps13*Δ mutant indicates that functional calcineurin is required. Interestingly, the *cmp2*Δ mutation caused cells not to be SDS-sensitive, and the double mutant *cmp2*Δ *vps13*Δ grew as single *cmp2*Δ (Figure 3c). Thus, the *cmp2*Δ mutation is epistatic to *vps13*Δ. Altogether, these results indicate that in the presence of SDS stress, the *vps13*Δ mutation triggers changes in the cell that can be overcome by the reduction of Cmp2 activity. This could be obtained by *RCN2* overexpression or deletion of the *CMP2* gene, but the active Cna1–Cnb1 phosphatase complexes are required.

### 2.4. Both rcn2-m1 and rcn2-m3 Mutations Abolish the Binding of Rcn2 to Catalytic Subunit of Calcineurin, Cmp2

**Figure 4:**
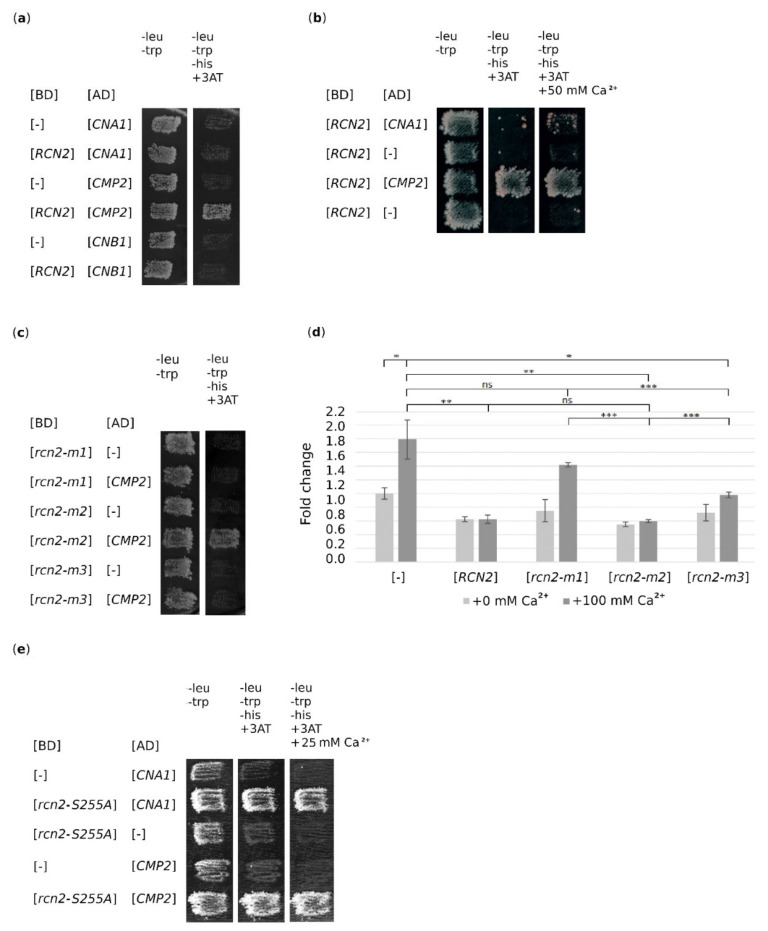
Rcn2 requires two motifs for the binding of Cmp2, and they are both required to efficiently inhibit calcineurin. (**a**) Rcn2 interacts with Cmp2, but not with Cna1 and Cnb1 in the two-hybrid system. (**b**) Addition of Ca^2+^ enables the interaction of Rcn2 with Cna1 in the two-hybrid system. (**c**) The *rcn2-m1* and *rcn2-m3* mutations abolished the interaction of Rcn2 with Cmp2. (**d**) Calcineurin is activated by calcium, and this effect is abolished by *RCN2,* but not by *rcn2-m1* overexpression. Calcineurin activity was assayed in wild-type cells transformed with empty plasmid [-] or plasmid bearing the indicated alleles of *RCN2*, which were grown in standard growth conditions (+ 0 mM Ca^2+^) or with the addition of calcium ions (+ 100 mM Ca^2+^). The graph presents means with error bars indicating SD. Results were analyzed using two-tailed Student’s t-test (n = 3, *p* < 0.0005 ***; <0.005 **; <0.05 *; ns, non-significant). (**e**) Rcn2-S255A interacts with both Cna1 and Cmp2 in the presence and in the absence of Ca^2+^.

We noticed that mutations in the *rcn2-m3* allele caused changes in the PSITVN sequence (aa 256–261) previously identified in the calcineurin binding site, described as PxIxIT-type [6,9], but did not abolish suppression. To clarify the mechanism by which *RCN2* suppresses *vps13*Δ mutant phenotypes, we tested the ability of mutant Rcn2 versions to bind to calcineurin subunits. By using the two-hybrid system, we tested whether we could detect the binding of Rcn2 to both phosphatase catalytic subunits, Cna1 and Cmp2 and, additionally, to the phosphatase regulatory subunit Cnb1. Using this approach, we found that Rcn2 bound to Cmp2, but not to Cna1 or Cnb1 (Figure 4a). As a control, we tested the interaction of Cna1 and Cmp2 with calmodulin (Cmd1), and we observed both interactions, as expected (Figure S3). The observed lack of interaction of Rcn2 with Cna1 was surprising, so we checked whether the addition of Ca^2+^ to the medium allowed the interaction of Rcn2 with Cna1. Indeed, in the presence of Ca^2+^ and after a long time (>7 days), the interaction was visible (Figure 4b), suggesting that the interaction of Rcn2 with Cna1 is probably weak and/or controlled. We also found that both Rcn2-m1 and Rcn2-m3 lost the ability to bind Cmp2 (Figure 4c) while Rcn2-m2 interacted with Cmp2 similarly to wild-type Rcn2. Thus, the interaction of Rcn2 with calcineurin required two different motifs of Rcn2: one at the N-terminus and the second, the PIxIxIT-type motif (aa 256–261) at the C-terminus. Moreover, the presence of this last motif alone was not sufficient to support interaction.

### 2.5. Partial Inhibition of Calcineurin Activity is Necessary for Suppression

To better understand the mechanism of *vps13*Δ suppression by Rcn2, we examined the influence of *RCN2* mutant gene overexpression on calcineurin activity. To compare the activity of calcineurin, we used a reporter system that comprised a plasmid containing a CDRE regulatory element fused to the *lacZ* open reading frame encoding β-galactosidase [21]. Upon calcineurin activation, the expression of CDRE element-driven sequences is upregulated, for example, in response to an increase in cytoplasmic calcium levels, resulting in an increased β-galactosidase activity. In our experimental settings, β-galactosidase activity was measured in cell extracts derived from transformants of the wild-type strain bearing empty vector or plasmids with different *RCN2* alleles. The transformants were grown in the absence or after the addition of extra calcium ions to induce calcineurin activity. The results demonstrate that the activity of calcineurin was slightly reduced by the overexpression of wild-type *RCN2* (p < 0.05) with an almost equivalent reduction by the expression of tested *rcn2* versions when cells were grown without the addition of calcium ions to the medium. As expected, when calcium ions were added to the culture of cells transformed with an empty vector, calcineurin activity increased (Figure 4d). The overexpression of the *RCN2* or *rcn2-m2* allele significantly prevented the activation of calcineurin under these conditions. The overexpression of the *rcn2-m1* allele did not affect calcineurin activity. The effect of the *rcn2-m3* mutation on Rcn2 action was minor compared with that of the *rcn2-m1* mutation but was significant. This indicates that two mutations, *rcn2-m1* and *rcn2–m3*, affect Rcn2 function as a calcineurin inhibitor; thus, at least two motifs in the Rcn2 protein determine its inhibitory activity toward calcineurin. One is the well-known calcineurin-binding motif PSITVN (aa 256–261) at the C-terminus. As revealed by studying Rcn2-m1, the second element that influences Rcn2-binding to calcineurin is found at the N-terminus, and this last element is necessary for the suppression of *vps13*Δ. Because the *rcn2-S255A* mutation also impairs the ability of Rcn2 to suppress *vps13*Δ and the resulting amino acid residue substitution is in the calcineurin-binding motif PSITVN (aa 256–261), therefore, we tested if this influences the interaction with Cna1 and Cmp2 in the presence and in the absence of Ca^2+^. Surprisingly, Rcn2-S255A interacted well with both alternative catalytic subunits (Figure 4e). This result supports the earlier conclusion that inhibition of only Cmp2-based activity is necessary for suppression.

### 2.6. Rcn2 Binds to the Cmp2 Fragment Corresponding to aa 382–455

**Figure 5:**
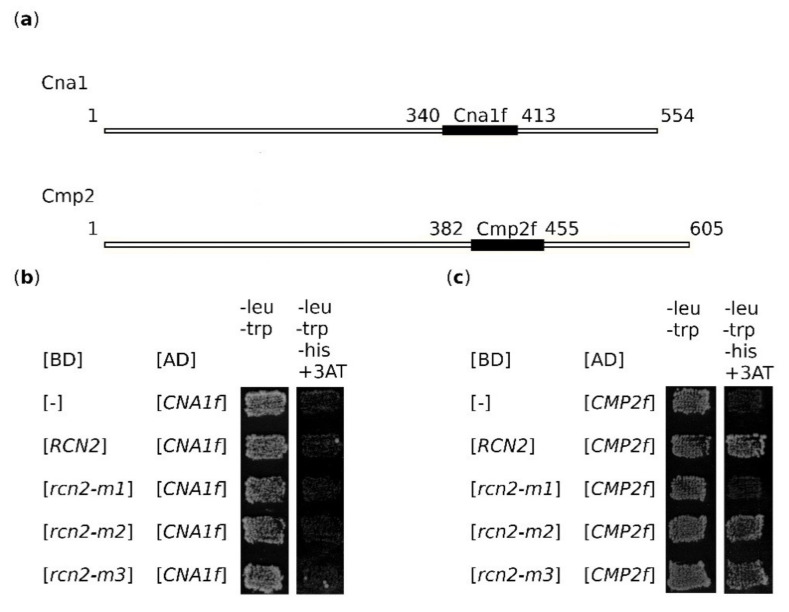
Rcn2 binds to the fragment of Cmp2 involved in the binding of the PXIXIT-type motif in substrates, but not to an analogous region of Cna1. (**a**) Schematic presentation of Cna1 and Cmp2 proteins with the indicated fragments (Cna1f; aa 340–413 and Cmp2f; aa 382–455) used to study interaction with Rcn2. (**b**) Rcn2 and Cna1f do not interact in the two-hybrid system. (**c**) Rcn2 and Cmp2f interact in the two-hybrid system. BD, DNA-binding domain; AD, activating domain.

Based on our results, we suspected that a second calcineurin-binding site might be present in the N-terminus of Rcn2. The Rcn1 inhibitor has two types of calcineurin-binding sites: the PxIxIT-type and LxVP-type [6]. Even though the calcineurin-binding site of the LxVP-type is not present in Rcn2, we speculated that Rcn2 might interact using an N-terminal motif that was inactivated in Rcn2-m1. We noticed that the sequence TQILIT (aa 9–14) in the Rcn2 protein is close to the PxIxIT consensus sequence, with only the P residue missing. For this reason, we investigated whether Rcn2 binds to the region of calcineurin catalytic subunits responsible for the binding of substrates. Fragments of Cna1 (Cna1f; aa 340–413) and Cmp2 (Cmp2f; aa 382–455) encompassing the sequence responsible for the binding of the PxIxIT-type motif (Figure 5a) were tested for interaction with Rcn2 using the two-hybrid system. The analysis revealed that Rcn2 and Rcn2-m2 and -m3 proteins do not interact with Cna1f (Figure 5b), but they do interact with Cmp2f, while Rcn2-m1 does not interact with either (Figure 5c). This result suggests that the N-terminal part of Rcn2 can indeed support binding to calcineurin even when the C-terminal PxIxIT-type motif (aa 256–261) is abolished. The second Cmp2-binding determinant of Rcn2 interacts with this phosphatase, although its aa sequence does not perfectly fit the consensus. Interestingly, the C-terminal PxIxIT-type motif of Rcn2 (aa 256–261) is not sufficient to support Cmp2-binding when the N-terminal motif is absent (*rcn2-m1*, Figure 5c). This result shows that the specificity of Rcn2 for binding the Cmp2 phosphatase subunit is maintained even when only a fragment of this protein is analyzed. In summary, Rcn2 binds a subset of calcineurin complexes, that is, those containing Cmp2, using two motifs—the already-described PxIxIT-type motif (aa 256–261) and the newly characterized motif, which encompasses aa 9–14 (TQILIT) at the N-terminus.

## 3. Discussion

Ca^2+^-ion signaling is fundamental for neuron function. Ca^2+^ fluxes across the plasma membrane and between intracellular compartments in response to different stimuli and calcium regulates several processes, such as neurite outgrowth, synaptogenesis, synaptic transmission, plasticity and cell survival. Vps13 proteins are also important for neuronal cell physiology and mutations in *VPS13* genes that cause genetic neurodegenerative disorders in humans. Using the yeast model of *VPS13*-related disorders, we have shown that calcineurin activity is potentiated in cells lacking the Vps13 protein and that the calmodulin/calcineurin signaling pathway could be a target for drug intervention in *VPS13*-dependent disorders. However, for the effective modulation of calcium signaling via the inhibition of calcineurin activity, the drug FK506 or the calcium chelator EGTA must be limited to a very specific concentration [30]. Here, using yeast, we found a different way to partially reduce the activity of calcineurin in *vps13*Δ cells and overcome the associated defects. We demonstrate that this could be achieved by overexpression of the *RCN2* gene encoding Rcn2 protein, a calcineurin inhibitor that binds preferentially to Cmp2, one of two alternative calcineurin catalytic subunits. Moreover, our studies reveal that binding between Rcn2 and Cmp2 requires two motifs in Rcn2: the already-known motif at the C-terminus and the newly characterized motif at the N-terminus. Because the Ca^2+^ signaling pathways are preserved from yeast to humans, we assume that our findings may be helpful for patients suffering from *VPS13*-dependent diseases or other neurodegenerative diseases characterized by high calcineurin activity, such as Parkinson’s disease.

Using yeast *vps13Δ* mutant cells, a model of ChAc and other *VPS13*-dependent diseases, we demonstrated that calmodulin–calcineurin signaling is a suitable target for drug intervention [30]. Similarly, for Parkinson’s disease, reduction of calcineurin activity was shown to mitigate alpha-synuclein toxicity in both yeast and mouse models [29]. Although drugs that reduce calcineurin activity, such as cyclosporin A and FK506, are known and have been used for years as immunosuppressants after transplantation, their use has many side effects on the nervous system, such as recurrent headaches, tremors, seizures, and even a decrease in cognitive function or changes in brain structure [8]. These symptoms put the long-term use of immunosuppressant drugs into question, especially for neurological disorders. This is also because both insufficient and excessive phosphatase activity has an adverse effect, as demonstrated in *vps13* mutant cells by us [30] or in alpha-synuclein-producing yeast cells by others [29]. Thus, further research is needed to discover alternative methods for the reduction of calcineurin activity without causing neurotoxicity, and studies in yeast can be very helpful [57]. Our multicopy suppressor screen was designed to find ways to circumvent the effects of the *vps13-I2749R* mutation, equivalent to a *hvps13A-I2771R* mutation found in ChAc patients [47], identified the *RCN2* gene encoding a negative regulator of calcineurin and gave us a hint for further study.

We tested two hypotheses about the molecular mechanism of Rcn2 action in the *vps13*Δ mutant. The first hypothesis assumes that overproduced Rcn2 binds and sequesters Slt2 and that this reduces Slt2 kinase activity toward other substrates, such as the plasma membrane high-affinity Ca^2+^ influx system comprising Cch1 and Mid1 proteins. As a consequence, decreased phosphorylation of the channel reduces its activity and calcium influx into the cell [27]. Our finding that the overexpression of the *rcn2-m2* allele, which encodes a protein variant unable to bind Slt2, is still able to suppress *vps13*Δ, argues against this hypothesis. In the same line is the finding that the homologous *RCN1* gene, which encodes the Rcn1 protein and also has potential Slt2-binding sites, did not rescue *vps13Δ* growth on SDS media. Although the study of the *rcn2-S255A* allele indicates that the S255 residue, an acceptor residue for Slt2-dependent phosphorylation, is important for the suppression of the *vps13*Δ mutant, it could be modified by another kinase instead. This can be concluded from the finding that the *rcn2-m2* allele, which encodes a protein that is not able to bind Slt2, is an efficient multicopy suppressor of *vps13*Δ. However, phosphorylation of residue S255 by unknown kinase is crucial for the regulation of Rcn2 interaction with alternative catalytic subunits of calcineurin.

The second most probable possibility for the mechanism of the suppression of *vps13* is that Rcn2 simply acts as a negative regulator of calcineurin. In fact, in studying various *rcn2* mutant alleles, we found that the ability to partially inhibit calcineurin is correlated with suppression. Using genetic analysis, we found that the *CNA1* and *CNB1* genes are required for *RCN2* suppression of *vps13*Δ and, importantly, that *cmp2*Δ alone is a suppressor of *vps13*Δ. Further analysis showed that Rcn2 interacts preferentially with a subset of calcineurin complexes, namely, those containing Cmp2 as a catalytic subunit. The weak interaction with Cna1 was only observed when cells were grown in the presence of additional Ca^2+^. This is in contrast to the earlier result of a co-immunoprecipitation experiment in which Rcn2 was found to interact equally well with both Cna1 and Cmp2 subunits [58]. The selective binding and inhibiting of the Cmp2 catalytic subunit (but not to Cna1), which is required for suppression, suggests the existence of a difference in specificity or regulation between Cmp2- and Cna1-containing calcineurin complexes. It was previously shown that the presence of the *CMP2* gene is more important than *CNA1* in the ER stress response [59]. It is worth noting that the activity of calcineurin is only partially inhibited by Rcn2, which is in agreement with the view that it might inhibit only a subset of calcineurin complexes. The observed selective interaction of Rcn2 with Cmp2 supports the hypothesis that the selective inhibition of Cmp2-based complexes is important for the survival of the *vps13*Δ mutant under stress conditions. This finding is additionally supported by the fact that Rcn2-S255A, which interacts with Cna1, lost the ability to suppress. In mammals, it is known that specific catalytic subunits of calcineurin also differ in their substrate specificity [60] and regulation. For example, it was shown that for T cell activation, calcineurin activity could be selectively regulated by ubiquitination and deubiquitination depending on the catalytic subunit [16]. The conclusion, similar to ours—that different RCANs have the potential to interact and regulate different calcineurin subunits isoforms—comes from studies in rodents in which three catalytic calcineurin subunits (CnA)—Aα, Aβ, and Aγ [61,62,63,64] have been shown to have different distributions in the brain corresponding to that of individual RCAN proteins [65]. Moreover, in humans, the distribution of calcineurin subunits is tissue-specific, with Aα and Aβ isoforms being ubiquitously expressed in a variety of tissues and Aγ restricted to the brain and testis [60]. Thus, it is possible that inhibiting one of the calcineurin isoforms in patients suffering from ChAc or other neurodegenerative diseases may help to overcome pathologies without the side effects connected to the inhibition of all isoforms by FK506.

The requirement for selective and only partial inhibition of phosphatase activity is also supported by the fact that *RCN1*, which encodes the other calcineurin regulator present in yeast cells, was not isolated in our screen and, when overexpressed, did not suppress the SDS hypersensitivity of the *vps13*Δ mutant. In fact, Rcn1 differs from Rcn2 in several ways. It interacts with Cna1 [6] and has the ability to activate calcineurin [9,66], but, most importantly, Rcn1 has two different types of calcineurin-binding motifs, the PxIxIT-type and LxVP-type, while in Rcn2, the LxVP-type motif is missing [58]. The presence of two phosphatase-interacting motifs in Rcn1 means that the binding is dual. This was demonstrated for RCAN1 or A238L from the African swine fever virus. Their motifs, similar to those in NFAT, occupy the PxIxIT-binding site on Cna, and the amino acid chain then turns back to allow the second motif to encompass the LxVP-binding pocket on the Cna/Cnb interface [67,68]. We demonstrate here that Rcn2 probably similarly interacts with Cmp2 using a dual-binding mode. This is suggested by the fact that Rcn-m3 is able to interact with Cmp2f, but not with Cmp2. Thus, the scenario is that Rcn2 first requires a C-terminal PSITVN motif (PxIxIT-type; aa 256–261) to bind Cmp2 and then the N-terminally located second motif. However, the difference is that the second motif in Rcn2 is not LxVP-like. This suggests that either Rcn2 possesses a structural calcineurin-binding motif in which part of the interacting surface is located at the C-terminus and the second part at the N-terminus, or there are two binding sites, and their binding occurs sequentially. The distinction between these two presented scenarios requires further study. In vitro studies have indicated that a synthetic peptide derived from the C-terminal part of Rcn2, the PSITVN motif, effectively competes with the PVIVIT peptide for binding to human calcineurin [9], which supports the idea of the presence of two independent binding motifs. This result also suggests that it should be possible to use a peptide derived from the N-terminal part of yeast Rcn2 to inhibit human calcineurin. Our present work indicates that this peptide may have a very desirable property. It has the potential for selective inhibition of only a subset of calcineurin and does not completely block calcineurin activity. For this reason, it should be less toxic for cells than immunosuppressant drugs such as FK506, similar to other tested peptides derived from human RCAN or the protein inhibitor A238L from the African swine fever virus [68].

Our studies are important from a clinical perspective. Here, we show a way to control calcium signaling more selectively than by the administration of calcineurin chemical inhibitors. Our present study suggests that new peptides with inhibitory activity toward calcineurin could be designed as an alternative to those investigated so far [68,69] and be specific to a subset of calcineurin complexes. Moreover, comparable changes observed on a molecular level, such as defects in vesicular trafficking, endocytosis, and the actin cytoskeleton, and increased calcineurin activity in yeast Parkinson’s alpha-synuclein model [29] and *VPS13*-deficiency model [46], which includes early-onset Parkinsonism, suggest that a similar approach can be taken for the treatment of several diseases.

## 4. Materials and Methods

### 4.1. Strains, Media, and Growth Conditions

For cloning and plasmid propagation, *E.coli* DH5α was used. The yeast strains used in this study are listed in Table S1 (Table S1). The strains KJK190 and KJK191 were constructed by integration of the *vps13::URA3* cassette amplified by PCR into the genomic DNA of the KJK181A strain. Strains BYcna1Δ and BYcmp2Δ were transformed with the PCR product, and transformants were selected on SC-Ura plates. Integrations were confirmed by PCR of genomic DNA. These strains were used to test the SDS sensitivity phenotype and the influence of *RCN2* overexpression on cell growth.

The media used were YPD complete medium (1% yeast extract, 2% peptone, 2% glucose) or synthetic SC medium (0.067% yeast nitrogen base without amino acids, 2% glucose) with supplements (adenine, uracil, amino acids) as indicated.

For growth tests, transformants were grown overnight in liquid media, and cultures were diluted to an optical density OD_600_ of ~1. Five-fold serial dilutions of cells were spotted on solid YPD with or without the addition of SDS (Sigma-Aldrich, St Louis, MO, USA) media, as described in the figure legends. Plates were incubated at 30 °C. The sensitivity of *vps13*Δ mutant cells to SDS is variable and depends on several factors, such as the medium lot, the lot of the SDS stock solution, and various physical factors; therefore, in each growth experiment, several SDS concentrations were tested, and one representative result is shown. For two-hybrid analysis, the PJ69-4A strain [70] was used, and respective transformants were grown on SC-Trp-Leu plates, replicated on SC-Trp-Leu-His supplemented with 1.5 mM 3-aminotriazole (Sigma-Aldrich) or SC-Trp-Leu-His supplemented with 1.5 mM 3-aminotriazole and 25 or 50 mM CaCl_2_. Plates were incubated at 30 °C for 3–10 days.

### 4.2. Plasmids, Mutagenesis, and Screen for Multicopy Suppressors of vps13 Mutations

All plasmids used in this study are listed in Table S2. Screening for multicopy suppressors of the *vps13-I2749R* SDS hypersensitivity phenotype is described in Soczewka et al. 2019 [30]. A plasmid with a DNA fragment containing the *RCN2* gene, which was isolated as a multicopy suppressor from the pFL44-based genome library [71], was used to amplify *RCN2* by PCR. Next, the amplified fragment was transferred to the YEp181lac plasmid [72] using BamHI and EcoRI restriction enzymes to obtain YEplac181-RCN2.

The *SLT2*, *CNA1*, *CMP2*, and *CNB1* genes were amplified by PCR using BY4741 genomic DNA as a template with appropriate primers (Table S3) and were cloned into pGBT9 and/or pGAD424 vectors. Mutant *rcn2-m1, rcn2-m2* and *rcn2-m3* alleles were obtained using one-step mutagenesis with appropriate primers. The construct YEp181lac-3HA-RCN2 contains a 3HA-tag coding sequence followed by the full-length *RCN2* gene. It is a derivative of the YEp181lac-KXS-RCN2 plasmid in which KpnI (K), XhoI (X), and SacI (S) restriction sites were introduced directly in front of the *RCN2* ATG sequence in YEp181lac-RCN2 by generating two types of overlapping PCR products (A and B) using Phusion High Fidelity DNA polymerase and the following pairs of primers. For the type A product, RCN2_For and L486KXS were used; for the B product, U486KXS and RCN2_Rev were used. Both products (A and B) were purified and used as templates for the next round of PCR reactions with the use of RCN2_For and RCN2_Rev primers. The final products were digested with BamHI and EcoRI, and the restriction fragments were transferred to the YEplac181-RCN2 plasmid, exchanging the original sequence for the introduced sequence with KpnI, XhoI, and SacI restriction sites immediately before the ATG codon. The 3HA encoding sequence was amplified from the plasmid pET9dSUMO [73] with primers 3HA-U50 and 3HA-L130 flanked by KpnI and SacI restriction sites. The PCR product was digested with KpnI and SacI and cloned into YEp181lac-KXS-RCN2. All PCR products were sequenced after cloning.

### 4.3. β-Galactosidase Activity Assay

β-Galactosidase activity was analyzed as previously described [74]. For this assay, cells were transformed with the pAMS363 plasmid bearing *CDRE-LacZ* fusion and either an empty plasmid or a plasmid bearing one of the *RCN2* alleles. Transformants were grown overnight in SC-Ura-Leu and then diluted and divided into two independent cultures, one of which was supplemented with CaCl_2_ to a final concentration of 100 mM. The cells were grown for another 4 h, and extracts were prepared using glass beads. In each of the three experiments, at least three independent transformants of the respective yeast strains were inoculated, and their extracts were assayed in duplicate.

### 4.4. Protein Extracts and Western Blot Analysis

Yeast cells were grown at 30 °C in SC-Leu to the logarithmic phase. Protein extracts were prepared by alkaline lysis [75]. Samples were analyzed by standard SDS–PAGE followed by Western blotting using a mouse monoclonal anti-HA epitope (BioLegend, San Diego, CA, USA) and Vma2 (Thermo Fisher Scientific, Waltham, MA, USA) or the polyclonal anti-GAL4 DNA-BD rabbit antibody (Sigma-Aldrich) followed by secondary anti-mouse or anti-rabbit IgG horseradish peroxidase (HRP)-conjugated antibodies (Dako, Glostrup, Denmark). The signal was detected by enhanced chemiluminescence (Millipore, Darmstadt, Germany).

## Data Availability

Data available on request from corresponding author due to privacy.

## Supplementary Materials

Supplementary Materials can be found at https://www.mdpi.com/1422-0067/22/3/1193/s1.
