# Supplementary material for: Partial Inhibition of Calcineurin Activity by Rcn2 as a Potential Remedy for Vps13 Deficiency
Source: Int J Mol Sci. 2021 Jan 26;22(3):1193. doi: 10.3390/ijms22031193 (PMC7865597; doi:10.3390/ijms22031193)
Supplement: Supplementary file 1 [file ijms-22-01193-s001.zip › Tables .docx]

**Table S1.** The list of strains used in this study.

| **Strain** | **Genotype** | **Source** |  |
| --- | --- | --- | --- |
| BY4741 | MAT**a** *his3*Δ*1*, *leu2*Δ*0* *met15*Δ*0 ura3*Δ*0* | Open Biosystem | |
| BYvps13Δ | MAT**a** *his3*Δ*1*, *leu2*Δ*0* *met15*Δ*0 ura3*Δ*0 vps13*::*kanMX* | Open Biosystem | |
| BYcna1Δ | MAT**a** *his3*Δ*1*, *leu2*Δ*0* *met15*Δ*0 ura3*Δ*0 cna1*::*kanMX* | Open Biosystem | |
| BYcmp2Δ | MAT**a** *his3*Δ*1*, *leu2*Δ*0* *met15*Δ*0 ura3*Δ*0 cmp2*::*kanMX* | Open Biosystem | |
| KJK181A | MAT**a** *his3*Δ*1* *leu2*Δ*0* *met15*Δ*0 ura3*Δ*0 vps13::URA3* | [60] | |
| PS3 | MAT**a** *his3*Δ*1* *leu2*Δ*0* *met15*Δ*0 ura3*Δ*0 cnb1*::*kanMX* | [30] | |
| PS4 | MAT**a** *his3*Δ*1* *leu2*Δ*0* *met15*Δ*0 ura3*Δ*0 cnb1*::*kanMX vps13::URA3* | [30] | |
| KJK190 | MAT**a** *his3*Δ*1*, *leu2*Δ*0* *met15*Δ*0 ura3*Δ*0 cmp2*::*kanMX vps13::URA3* | This work | |
| KJK191 | MAT**a** *his3*Δ*1*, *leu2*Δ*0* *met15*Δ*0 ura3*Δ*0 cna1*::*kanMX* *vps13::URA3* | This work | |
| PJ69-4A | MAT**a** *trp1*Δ*-901 leu2-3,112 ura3-52 his3-*Δ*200 gal4*Δ *gal80*Δ *GAL2-ADE2 LYS2::GAL1-HIS3 met2::GAL7-lacZ* | [77] | |

**Table S2.** The list of plasmids used in this study.

| **Plasmid** | **Source** |
| --- | --- |
| pGBT9 | Clontech |
| pGAD424 | Clontech |
| pGAD424-SLT2 | This study |
| pGAD424-CMP2, | This study |
| pGAD424-CMP2-f | This study |
| pGAD424-CNA1 | This study |
| pGAD424-CNA1-f | This study |
| pGAD424-CNB1 | This study |
| pGBT9-RCN2 | This study |
| pGBT9-rcn2-m1 | This study |
| pGBT9-rcn2-m2 | This study |
| pGBT9-rcn2-m3 | This study |
| YEp181lac | [79] |
| YEp181lac-RCN2 | This study |
| YEp181lac-rcn2-m1 | This study |
| YEp181lac-rcn2-m2 | This study |
| YEp181lac-rcn2-m3 | This study |
| YEp181lac-rcn2-S255A | This study |
| YEp181lac-KXS-RCN2 | This study |
| YEp181lac-3HA-RCN2 | This study |
| YEp181lac-3HA-rcn2-m1 | This study |
| YEp181lac-3HA-rcn2-m2 | This study |
| YEp181lac-3HA-rcn2-m3 | This study |
| pRK109S | [61] |
| pET9dSUMO | [80] |
| pAMS363 | [21] |

**Table S3.** The list of oligonucleotides used.

| **Primer** | **Sequence** |
| --- | --- |
| SLT2-1 | 5’ GCCGTCGACCTTCCCCGGTTACTTATAGT 3’ |
| SLT2-2 | 5’ CCCGAATTCATGGCTGATAAGATAGAGAG 3’ |
| RCN2-1 | 5’ CGGGTCGACGCTCTAGTGCTCTAATGGA 3’ |
| RCN2-2 | 5’ CGCGAATTCATGGCAAACCAAAAGCAAAT 3’ |
| CNA1-1 | 5’ AACCCGGGGATGTCGAAAGACTTGAATT 3’ |
| CNA1-2 | 5’ TTCTGCAGTCACAGTTGTGGCTTTTTC 3’ |
| CMP2-1 | 5’ CCGAATTCACCCTTTTCAGTGCGCCTAA 3’ |
| CMP2-2 | 5’ CCGTCGACCAGCTCATCTTCAGTACAGA 3’ |
| CNB1-1 | 5’ AAGAATTCAATGGGTGCTGCTCCTTCCA 3’ |
| CNB1-2 | 5’ AAGGATCCTTACACATCGTATTGCAAT 3’ |
| RCN2_For | 5' AAAGGATCCGCTTCTGTCGGTCTAATCTC 3' |
| RCN2_Rev | 5' AAAGAATTCTAAGTGTTCAAGCAGGACTGT 3' |
| RCNM1-1 | 5’ TGTCCGTCGCGAGTGCTTGAGTTCTCATTTGCTTTTGG 3’ |
| RCNM1-2 | 5’ CACTCGCGACGGACATTCCTAGTGG 3’ |
| RCNM2-1 | 5’ AGATGCTGCAGGCGCGTTGCCATGATCCTCCAAAA 3’ |
| RCNM2-2 | 5’ CGCCTGCAGCATCTATTAATACAGATCCAGGAGTCACTGG 3’ |
| RCN2M3-1 | 5’ CTGCCGCGGCTGGACTTTTTGGAGGATTTG-3’ |
| RCN2M3-2 | 5’ GTCCAGCCGCGGCAGCGAACGAGTTTTTCCATTAGAGC-3’ |
| U486KXS | 5' GGTACCCTCGAGGAGCTCGCAAACCAAAAGCAAATGAGA 3' |
| L486KXS | 5' GAGCTCCTCGAGGGTACCCATAATTCCCGATTTTCTAAC 3' |
| 3HA-U50 | 5’ GGGGTACCTACCCATACGATGTTCCT 3’ |
| 3HA-L130 | 5’ CCGAGCTCAGCGTAATCTGGAACGTC 3’ |
| RCN2-S255AU | 5’ AAGCACCCAGCATAACGGTTAACGAGT 3’ |
| RCN2-S255AL | 5’ ATGCTGGGTGCTTTTGGAGGATTTGAG 3’ |
| CNA1F-U | 5’ AAGAATTCACCATGTTCAGTGCGCCAAA 3’ |
| CNA1F-L | 5’ CCGTCGACAAGTTCCTGCTCACTACATA 3’ |
| CMP2F-U | 5’ CCGAATTCACCCTTTTCAGTGCGCCTAA 3’ |
| CMP2F-L | 5’ CCGTCGACCAGCTCATCTTCAGTACAGA 3’ |
