# Supplementary figures and images for: Partial Inhibition of Calcineurin Activity by Rcn2 as a Potential Remedy for Vps13 Deficiency
Source: Int J Mol Sci. 2021 Jan 26;22(3):1193. doi: 10.3390/ijms22031193 (PMC7865597; doi:10.3390/ijms22031193)

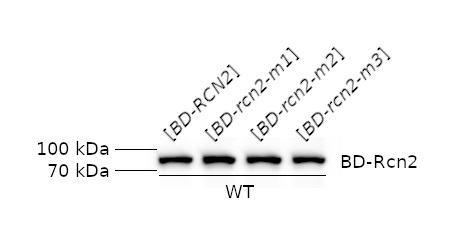

Supplement: Supplementary file 1 [file ijms-22-01193-s001.zip › S1.jpg]

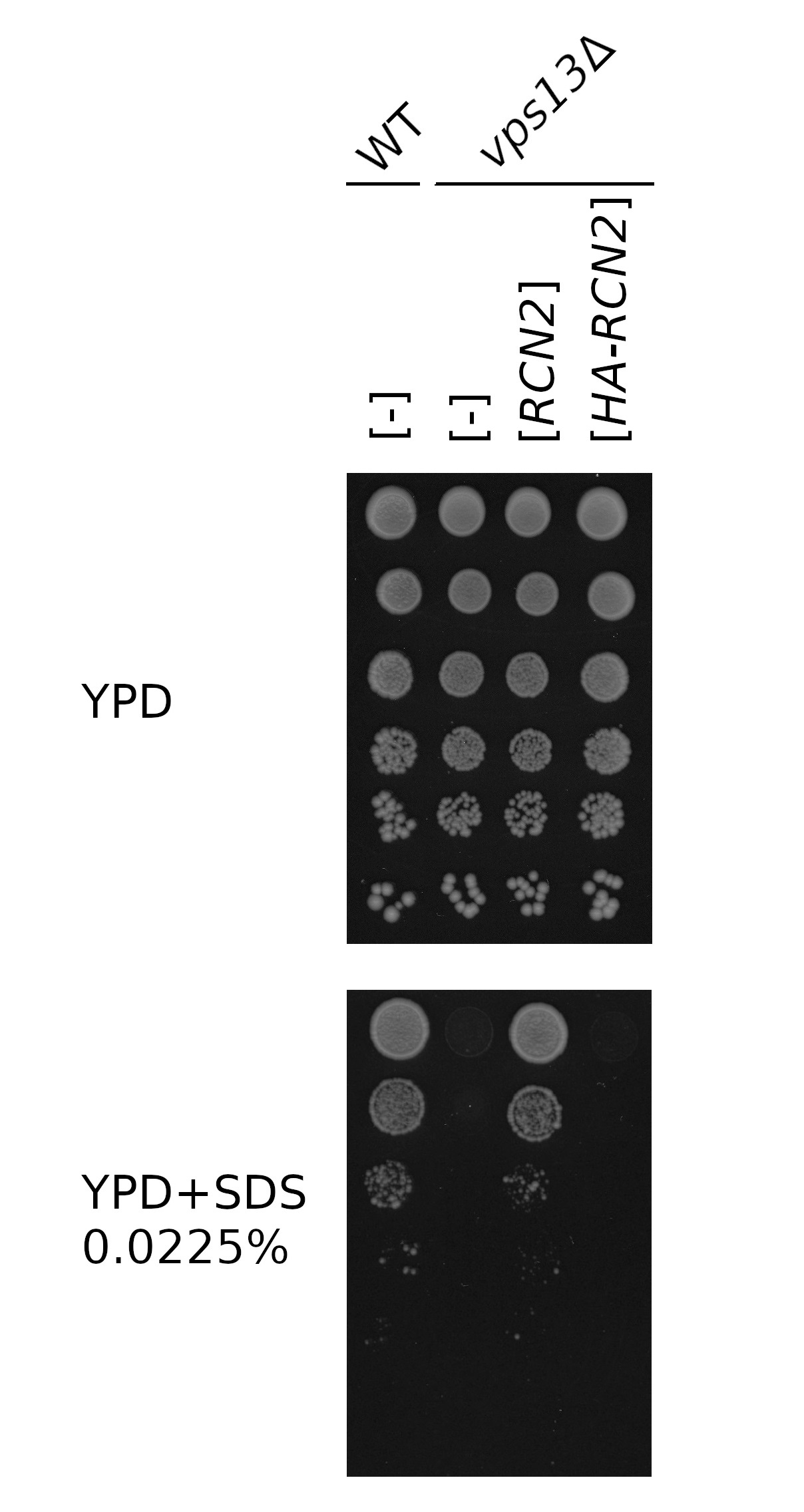

Supplement: Supplementary file 1 [file ijms-22-01193-s001.zip › S2.jpg]

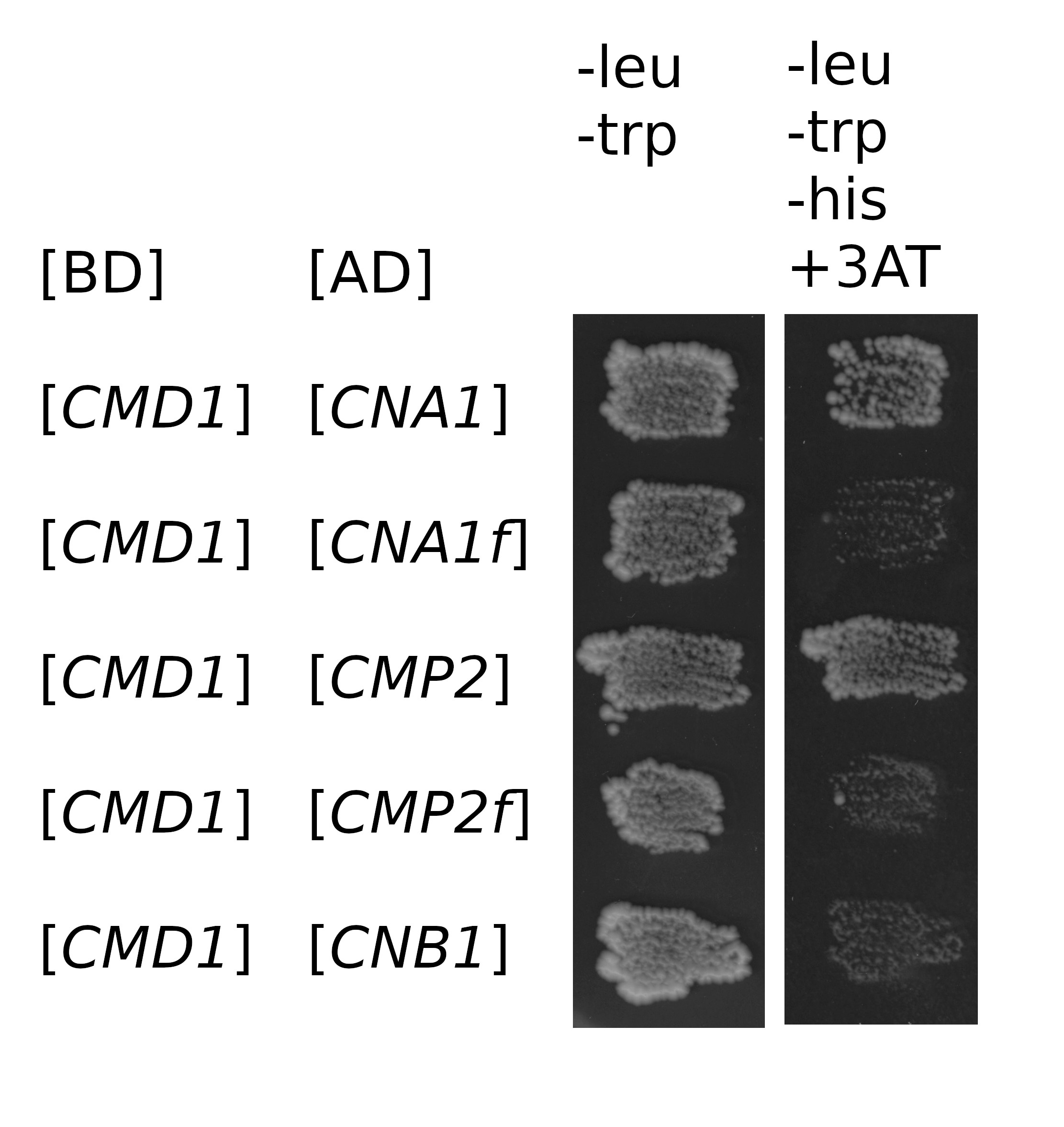

Supplement: Supplementary file 1 [file ijms-22-01193-s001.zip › S3.jpg]
